# A Qualitative Exploration of Patient and Clinician Views on Patient Reported Outcome Measures in Child Mental Health and Diabetes Services

**DOI:** 10.1007/s10488-014-0586-9

**Published:** 2014-09-02

**Authors:** Miranda Wolpert, Katherine Curtis-Tyler, Julian Edbrooke-Childs

**Affiliations:** 1 Policy Research Unit in the Health of Children, Young People and Families, Evidence Based Practice Unit, UCL and Anna Freud Centre, 21 Maresfield Gardens, London, NW3 5SD UK; 2 School of Health Sciences, City University, London, UK

**Keywords:** Patient Reported Outcome Measures (PROMs), Mental health, Diabetes, Qualitative

## Abstract

Patient Reported Outcome Measures (PROMs) are increasingly being recommended for use in both mental and physical health services. The present study is a qualitative exploration of the views of young people, mothers, and clinicians on PROMs. Semi-structured interviews were conducted with a sample of *n* = 10 participants (6 young people, 4 clinicians) from mental health services and *n* = 14 participants (4 young people, 7 mothers, 3 clinicians) from a diabetes service. For different reasons, young people, mothers, and clinicians saw feedback from PROMs as having the potential to alter the scope of clinical discussions.

## Introduction

The routine use of Patient Reported Outcome Measures (PROMs) is recommended by healthcare policy for a range of long-term conditions in the United Kingdom (UK), including services for young people with mental health problems or diabetes (Department of Health 2011, 2012; Royal College of Paediatrics and Child Health 2012). PROMs have been defined elsewhere in this special issue (Edbrooke-Childs et al. 2014). In child mental health services, PROMs are being implemented to measure change in mental health symptoms and functioning (e.g., Revised Children’s Anxiety and Depression Scales: Chorpita et al. 2005) and to measure progress to achieving therapeutic goals (i.e., Goal Based Outcome measure: Law 2011; Wolpert et al. 2012). In child diabetes services, PROMs are being implemented to measure change in general wellbeing (e.g., Pediatric Quality of Life Inventory (PedsQL) Varni et al. 1999; Royal College of Paediatrics and Child Health 2012).

Evidence suggests that the routine use of PROMs may benefit shared treatment decision making between patients and clinicians, service satisfaction, and treatment progress monitoring (Batty et al. 2013; Bickman et al. 2011; De Wit et al. 2006; Skevington and McCrate 2012; Wolpert 2013). On the one hand, research has focussed on the development of PROMs and ensuring they measure salient issues to patients (Skevington and McCrate 2012). On the other hand, there is little research exploring patient and clinician attitudes to PROMs (Batty et al. 2013).

As reported elsewhere in this special issue (de Jong 2014; Douglas et al. 2014; Edbrooke-Childs et al. 2014; Fleming et al. 2014; Mellor-Clark et al. 2014), there are a number of barriers to implementing PROMs, mirrored in qualitative studies with patients and clinicians. Support is indeed needed for services to overcome these barriers and routinely use PROMs. As demonstrated in an audit of a child mental health service, the routine administration of PROMs doubled after one year, with the support of an active learning collaboration (Hall et al. 2013).

Barriers to using PROMs regard three main areas (Badham 2011; Batty et al. 2013; Martin et al. 2011; Moran et al. 2012; Norman et al. 2013; Stasiak et al. 2013).

Firstly, the content and format of measures. This includes their structured format, the focus on problems or deficits, and the inability to capture the complexity of the young person’s experience and context.

Secondly, the process of using measures, as these may be perceived to interfere with therapeutic engagement.

Finally, the outcome of using measures, considering whether data obtained from measures may be more relevant and useful to services than to patients.

A particular issue for the outcome of using measures is that patients may be unwilling to reveal some topics or may feel that PROMs do not capture their concerns and priorities. Furthermore, clinicians may not view problems identified using PROMs as warranting a change in practice or referral because they may be seen as either an inevitable side effect of treatment or, on the contrary, as not being caused by treatment or presenting problems (Greenhalgh 2009; Marshall et al. 2006).

Young people have suggested that the use of PROMs may be encouraged by making measures more convenient and flexible, promoting ‘quick’, ‘simple’, ‘well-explained’, and ‘optional’ measures (Badham 2011, p. 13). How PROMs are used rather than the measures themselves may be a key determinant of attitudes, and young people appear to support PROMs when used after a rapport has been developed with the clinician (Stasiak et al. 2013). Clinicians report concerns around resourcing and a lack of time for and ownership of implementation, some of which may be offset by computerised implementation to ensure timely feedback to clinicians (Batty et al. 2013; Martin et al. 2011; Murphy et al. 2011).

Given the increasing use of PROMs in services for young people (Department of Health 2011, 2012; Royal College of Paediatrics and Child Health 2012), research is required to understand young people’s, parents’, and clinicians’ views on PROMs. In particular, understanding perspectives on the barriers and facilitators to implementing PROMs, and how these may be similar and different across mental and physical healthcare settings, may help inform how to appropriately tailor implementation.

### Aims of the Present Research

To the best of our knowledge, no study has explored the similarities and differences in perspectives of patients and clinicians in settings related to long-term mental and physical conditions in young people. To this end, the aim of the present research was to explore the views of young people, parents, and clinicians from child mental health and diabetes services to understand their views on PROMs, the implementation of PROMs, and the barriers to implementation.

## Method

### Participants

Semi-structured interviews (see Appendix 1) were conducted with a sample of *n* = 10 participants (6 young people and 4 clinicians) from child mental health services and *n* = 14 participants (4 young people, 7 mothers, and 3 clinicians) from a child diabetes service; young people in the diabetes service all had type 1 diabetes. Further demographic characteristics of the sample are shown in Table 1.

**Table 1:** Sample characteristics

Participants (*n*)	Interview type	Young people				Mother’s ethnicity (*n*)
		Gender (*n*)	Age (*n*)	Ethnicity (*n*)	Years accessing services (*n*)	
Young people from child mental health services (6)	Face-to-face in focus group (6)	Male (5), female (1)	13–17 (6)	White British (4) Mixed Ethnic Group (2)	1 (2) 4–7 (2) 10 (1) Unknown (1)	Unknown (6)
Young person-mother dyads from diabetes services (4)	Face-to-face (4)	Male (2), female (2)	9–10 (3) 13-17 (1)	White British (3) Mixed Ethnic Group (1)	1 (2) 4–7 (1) 10 (1)	White British (3) White Other (1)
Mothers from diabetes services; child not interviewed (3)	Individually face-to-face (2) or by phone (1)	Male (1), female (2)	0–5 (3)	White British (2) Mixed Ethnic Group (1)	1 (3)	White British (2) White Other (1)

### Procedure

Young people and mothers were recruited using advertisements with voluntary sector organisations. Clinicians were recruited using the research team’s networks of clinicians leading work to introduce PROMs. Interviews took place over 6 months during 2011 and were conducted by a senior qualitative researcher (KCT), either by telephone or face-to-face (see Table 1 for details). Interviews were conducted individually, in child-mother dyads, or in a focus group, which was held with members of a child mental health service user participation group.

In all interviews, participants were asked about the information clinicians should seek in order to understand patients’ priorities for care and about their views on the implementation of PROMs. Discussions were recorded and transcribed, then, using the constant comparative method, data were coded on themes from previous studies and those arising directly from the accounts of participants in this study. The data was summarised in charts by case and theme to facilitate identification of patterns (and disconfirming cases) within and across cases (Ritchie et al. 2003; Glaser and Strauss 1967).

Young people and their mothers had not routinely used PROMs in their treatment, as the study was conducted before PROMs had been implemented in these settings. Hence, in child mental health services, participants were asked for their views on a symptom checklist currently used in these services (Jones et al. 2013); in the diabetes service, participants were asked for their views on the PedsQL (Varni et al. 1999) currently used in these services.

### Ethical Approval

The study was approved by the School of Health Sciences Research Ethics Committee at City University, London. The study was conducted in line with the British Psychological Society Code of Human Research Ethics (BPS 2010) and all participants gave written informed consent prior to participation. On meeting the participants, the researcher emphasised that participation was voluntary and that they could stop taking part at any point without giving a reason; no one withdrew. Young people and mothers were given a £10 voucher as a thank you for participation.

### Data Analysis

All interviews were audio-recorded and transcribed verbatim. Data were then summarised and explored using thematic analysis, by comparing patterns, similarities, and differences across cases. Data analysis was performed by the second author (KCT). Primary and secondary themes are explored, below.

## Results

### Scope of Clinical Discussions

Clinicians across both settings saw PROMs as a tool for altering the scope of clinical discussions, in turn creating opportunities to tailor care more closely to individual need. Most envisaged implementing PROMs alongside measures of patient experience and, as recommended (Greenhalgh 2009), individualised goal setting. Some clinicians felt that asking patients for their opinion might be partially empowering.

In a context where problems and ‘possible ways forward’ may be less than clear cut (Clinician 1), mental health clinicians hoped that PROMs might enable interventions to be more tightly focused on the needs of the family, promoting ‘better, quicker outcomes’. Here, there was a drive to hear families’ perspectives and priorities in the first appointment, where they might be offered ‘two or three choices’ for intervention (Clinician 1) and progress could then be monitored using PROMs at every session. However, young people saw this focussing of treatment as potentially threatening to the patient–clinician relationship (see ‘pace of clinical discussions’ below).

#### Stigma

Young people and mothers in the diabetes service worried that widening the scope of clinical discussions, through PROMs, would attract unwarranted professional attention to emotional issues, potentially opening families up to stigma associated with mental health problems. Mothers also feared professional scrutiny of the quality of care they were providing for their children, if emotional issues were raised.

#### Communication

Young people with diabetes reported that describing their experiences in the clinic could be a challenge: ‘I have to think about [what I say] for a very long time and then I can’t be accurate with my words’ (Young Person 1, 14, diabetes). There were mixed feelings as to whether this would be supported by the use of PROMs. For instance, Young Person 2 (9, diabetes), reported some confidence that the PROM might help her ‘show what I feel like… so [clinicians] can help me,’ while Young person 1 disagreed: ‘I put it down because it… matched how I felt but I’m not sure how to approach it now I have written it.’

### Patient–Clinician Relationships

Clinicians and service users echoed concerns voiced in previous studies (Moran et al. 2012; Norman et al. 2013; Stasiak et al. 2013) about the potential for PROMs to impact negatively on the patient-clinician relationship.

#### Safe to Reveal to Clinicians

Young people and mothers in both settings expressed uncertainty about what PROMS would be used for, and on the basis of this, what would be appropriate, or safe, to reveal to clinicians (Stasiak et al. 2013). Young people were concerned about what their answers might reflect on their clinician and his or her practice: ‘I wouldn’t know if it would be rude to tick like ‘worse’ or ‘much worse’ (Young Person 3, 14, CAMHs). Young people in mental health services were concerned that PROMs might be used to narrow access to care; e.g., ‘If I tick ‘much worse’ then I don’t know if [that means] “I don’t need this service”, I don’t know, I’m very confused,’ (Young Person 4, 14, CAMHS). What is safe to reveal with PROMs may reflect a general fear of the unknown that children and their parents report when first attending CAMHS (Bone et al. 2014).

Similarly, clinicians from both settings felt that it would be important to provide patients with a good rationale for PROMs, explaining how information would be used, and to provide feedback from information obtained from PROMs.

#### Pace of Clinical Discussions

Unlike clinicians in mental health services who viewed PROMs as useful to immediately target interventions, young people wanted to use the first few sessions ‘to build a rapport instead of going straight into the nitty–gritty,’ (Young Person 3, 14, CAMHS).

If he just suddenly asks you, ‘So tell me about your problem,’ you wouldn’t just say, like, ‘Okay’. You’d tell your best friend or someone you are really, really close to but you wouldn’t tell this random person you’d just met’ (Young Person 3, 14, CAMHs).

In contrast, mothers from the diabetes service were worried that changing the scope of clinical discussions to encompass emotional problems raised by PROMS might detract from time available to discuss issues relevant to diabetes management or the ‘objective hardcore,’ (Mother 2).

### Tension Between the Fixed and the Fluid

Clinicians from both settings reported a ‘real tension’ (Clinician 1) between the fixed instruments and the fluidity patients’ unique experiences. Chiming with this, young people and mothers showed concern about how to represent their individual experiences within the fixed structures of the measures (Moran et al. 2012).

The thing is, I think that the reason I wouldn’t want to do it beforehand, is because I might start doubting whether my answers were right…if I do it quickly, on the paper, I’ve done it and I can’t go back and change it all. (Young Person 5, 10, Diabetes).

### Practicalities

Clinicians suggested that PROMs should be made more usable, dovetailing with previous studies (Norman et al. 2013); e.g., ‘something relatively uninvasive, something fairly quick…something much more easily useable for the everyday clinician,’ (Clinician 2). Clinicians also highlighted the need for extensive information technology and administrative support for implementation, especially to ensure timely feedback of results to clinicians and young people: ‘what happens in a week could very much change your life quite quickly as a teenager’ (Clinician 7).

### Appeal

Across settings, young people and mothers shared a view that measures could be improved by using a more unstructured, flexible format and by making them more visually appealing with electronic administration. Some suggested using pictures and symbols instead of numbers and words. Young Person 6 remembered a CAMHS worker using a chart where ‘there was loads of different people sat up in a tree and loads of different faces and you… circle the one you’re feeling’. Young Person 2’s mother (diabetes) suggested: ‘it would be quite interesting to almost do it pictorially…you move your needle-pricker along a sliding scale.’

### Service Development

Some clinicians valued standardised tools to facilitate audit and service development.

[Benefits of] the outcome monitoring [for service development]… become very slightly further away from the client, and then get back to them. So if you find that we’re not particularly good at eating disorders, then it may have an impact on sort of training in the service, that we get better at doing eating disorders, and then eventually a cohort of clients that come through get a better service (Clinician 1).

## Brief Discussion

The aim of the present research was to explore the views of young people, parents, and clinicians from child mental health and diabetes services to understand their views on PROMs, the implementation of PROMs, and the barriers to implementation.

For different reasons, young people, mothers, and clinicians viewed PROMs as having the potential to alter the scope of clinical discussions. On one hand, clinicians reported this as being an opportunity to better tailor care to individual need and to do so from the very start of treatment. On the other hand, young people in mental health services reported concerns that this would alter the pace of clinical discussions, prioritising a focus on treatment at the expense of therapeutic relationship building. Young people and mothers in the diabetes service were concerned about clinical discussions moving away from physical to mental health concerns, bringing with it increased stigma and scrutiny. Young people and mothers from both settings raised concerns about how data obtained from PROMs would be used and what would be safe to reveal using measures. In particular, young people from mental health services were concerned that responses would be used to narrow access to services.

Other potential barriers were information technology and administrative support and the tension between the fixed structure of PROMs and the need to capture the fluidity of patients’ experiences. Still, some clinicians valued the capacity of structured instruments to draw comparisons of outcomes across patient groups, which it was hoped would facilitate service development.

Limitations should be considered when interpreting the findings of the present research. First, the sample was small, and conclusions may not generalise beyond the present sample. Second, participants were recruited using the research team’s professional networks and advertisements, which risk self-selection bias. Finally, interviews with young people with diabetes and their mothers were conduct in dyads, potentially limiting young people’s freedom of expression.

Notwithstanding the above limitations, the present research is the first to explore and triangulate the views of young people, mothers, and clinicians from child mental health and diabetes services to understand their views on PROMs, the implementation of PROMs, and barriers to implementation.

Findings from the present research highlight the need for well-planned and resourced support for PROM implementation, including guidelines to help clinicians know how to administer and interpret PROMs (Devlin and Appleby 2010; Greenhalgh 2009; Greenhalgh et al. 2005; Law 2012). As mentioned by clinicians from both settings, young people and their parents need explanations of the rationale for using PROMs, as they may have a range of concerns about the content, process, and outcome of implementing measures. If clinicians are to use PROMs to guide practice with their patients, they may need to find ways to foster young people’s and parents’ ownership of measures and data. Using PROMs within a shared decision making context may help achieve this (Law 2012; Wolpert 2013).

To do so, clinicians should discuss with young people and parents when to use- and not use-measures. If it is decided to use measures, discussions should explore which areas to focus on in sessions and correspondingly, what measures to use. The potential clinical utility of measures should be considered in terms of monitoring and reviewing treatment progress. Such data obtained from PROMs can be used to look for off track cues so clinicians can discuss with young people and their parents instances when treatment might not be progressing as expected. Action plans can then be devised to try new methods to redress treatment progress, with PROMs used to continue to monitor the impact of changes.

Findings of the present research suggest that young people, mothers, and clinicians might all view PROMs as having the potential to alter the scope of clinical discussions. These alterations need to be carefully discussed with young people and mothers as they may view potential alterations differently to clinicians. Clinicians in the present study viewed PROMs as potentially useful for tailoring care to the needs of the patient from the outset of treatment. However, young people in mental health services were concerned that this may take time away from therapeutic relationship building in the initial stages of treatment. Conversely, young people and their mothers in the diabetes service were concerned that emotional issues raised by PROMs may detract from physical healthcare.

For different reasons, young people, mothers, and clinicians in both mental and physical healthcare viewed PROMs as having the potential to alter the scope of clinical discussions. Before implementing PROMs, clinicians need to be aware of the associated strengths and barriers this may bring, so they can carefully communicate these to the young people and parents in their care.

## Appendix 1: Questions Used with Young People and Carers and with Clinicians

### For Young People and Carers

- The government is very keen for young people, carers and clinicians to work together closely to support young people’s health. What kinds of things matter to you about your health? (Offer young people opportunities to make drawings).
- What kind of things do you think different clinicians should be asking you and your families/children when you meet? (Offer young people opportunities to make drawings).
  - Why? How should they do this? (Prompt regarding different mediums and times e.g. online, groups etc.)
- At the moment, what different kinds of information do different clinicians ask you and your families/children for when you meet? (prompt regarding physiological data, as well as other kinds).
  - Why do they need this? What do they do with it, or what do they use it for?
  - How does it all fit together?
  - Do you think this helps you work closely with clinicians or do you think other approaches would be useful?
- (If young people/carers don’t currently have experience of PROMS):
  - PROMS are sets of questions which clinicians ask patients, often each time they meet, in order to measure their well-being (show example). The government is keen for clinicians to use these in their work with young people and their families.
  - What do you think you might feel answering questions like these when you met your clinician?
  - How could clinicians ask these questions in ways that work well for you?
  - Or is there a different way clinicians could gather this information that you would prefer? (Prompt online, in group work etc.)

### For Clinicians

- The government is very keen for young people, carers and clinicians to work together closely to support young people’s health. In order to do this, what kind of things do you think different clinicians should be asking young people and their families when they meet?
  - Why?
  - How should they do this? (Prompt regarding different mediums and times e.g. online, groups).
- At the moment, what different kinds of information do different clinicians ask young people and their families for when they meet? (prompt regarding physiological data as well as other kinds).
- Why is this?
- What do you do with the information or what is it used for? How do the different types of information fit together?
- Do you think this helps you work closely with young people and families or do you think other approaches would be useful?
- What was the rationale for developing PROMS in your setting?
  - What is the timetable for the development of the PROM?
  - What outcomes do you anticipate from introducing PROMS?
  - How will these be achieved? (Prompt regarding each step of mechanism).
  - How will these work across the patient-carer-clinician triad and within the wider multi-disciplinary team?
- There is some evidence from CAMHS settings that young people and some clinicians have negative views of PROMS: what impact do you think PROMS will have on your relationships and working with young people and families?
  - How can they be implemented in ways that support collaborative working across the patient-carer-clinician triad? Prompt regarding different way clinicians could gather this information e.g. online, in group work etc.
